# *A1BG-AS1* promotes adriamycin resistance of breast cancer by recruiting IGF2BP2 to upregulate ABCB1 in an m6A-dependent manner

**DOI:** 10.1038/s41598-023-47956-2

**Published:** 2023-11-25

**Authors:** Jian Wang, Jie Xu, Jie Zheng

**Affiliations:** 1 Department of General Surgery, Tianjin Fifth Central Hospital, No. 41 Tanggu Zhejiang Road, Binhai New Area, Tianjin, 300450 China; 2 Department of Pathology, Tianjin Fifth Central Hospital, Tianjin, 300450 China

**Keywords:** Cancer, Molecular biology, Biomarkers, Oncology

## Abstract

Adriamycin (ADR) resistance is an obstacle for chemotherapy of breast cancer (BC). ATP binding cassette subfamily B member 1 (ABCB1) expression is indicated to be closely related to the drug resistance of cancer cells. The current work intended to explore the molecular mechanisms to regulate ABCB1 in BC cells with ADR resistance. We found that long noncoding RNA (lncRNA) A1BG antisense RNA 1 (*A1BG-AS1*) is upregulated in ADR resistant BC cell lines (MCF-7/ADR, MDA-MB-231/ADR). *A1BG-AS1* knockdown enhanced the ADR sensitivity by suppressing the viability, proliferation potential and migration ability, and facilitating cell apoptosis in BC. Insulin-like growth factor 2 mRNA-binding protein 2 (IGF2BP2) is known to be an m6A reader to modulate the stability of mRNA transcripts in an m6A-dependent manner, which was a shared RNA binding protein (RBP) for *A1BG-AS1* and ABCB1. The interaction of IGF2BP2 with *A1BG-AS1* or ABCB1 was explored and verified using RNA pulldown and RNA immunoprecipitation (RIP) assays. ABCB1 mRNA and protein expression was positively regulated by *A1BG-AS1* and IGF2BP2 in BC cells. ABCB1 mRNA expression was stabilized by *A1BG-AS1* via recruiting IGF2BP2 in an m6A-dependent manner. Moreover, rescue assays demonstrated that *A1BG-AS1* enhanced BC ADR resistance by positively modulating ABCB1. Xenograft mouse models were used to explore whether *A1BG-AS1* affected the ADR resistance in BC in vivo. The findings indicated that *A1BG-AS1* silencing inhibited tumor growth and alleviated ADR resistance in vivo. In conclusion, *A1BG-AS1* enhances the ADR resistance of BC by recruiting IGF2BP2 to upregulate ABCB1 in an m6A-dependent manner.

## Introduction

Breast cancer (BC) accounts for 30% of female cancers, with increasing incidence, remains a challenge to human health^1^. Over 2 million new cases of BC are reported in 2020, representing 11.7% of all cancer cases^2^. Many risk factors may contribute to BC, including the pregnancy-associated factors, lifestyle factors such as obesity and smoking, age and genetic mutations^3^. Surgery, radiotherapy and chemotherapy are the main treatment options for BC patients, while the recurrence rate is still high^4^. ADR is the first line drugs used for BC treatment, and the drug resistance remains a challenge for anti-cancer therapies.

ABCB1 (ATP binding cassette subfamily B member 1, also named MDR1) present in the cell membrane encodes P-glycoprotein (P-gp), and regulates the distribution, absorption and excretion of various chemical compounds. P-gp is indicated to reduce the intracellular drug accumulation and P-gp upregulation is closely correlated with the enhanced drug resistance to targeted chemotherapy^5–7^. For example, in neuroblastoma, MDM2-p53 antagonists could act as modulators of MDR1 to maintain high intracellular concentration of vincristine (VCR)^8^. Currently, numerous studies have reported the effects exerted by ABCB1 on BC ADR resistance. For example, ABCB1 is highly expressed in chemoresistant BC tissues and ADR resistant cells. ABCB1 alleviates the effect of ADR by pumping drugs out of cancer cells^9^. ABCB1 mediates ADR resistance in BC, which is regulated by the growth arrest-specific 5 (GAS5)/miR-221-3p/(dickkopf 2)DKK2 axis^10^. The activation of ABCB1 by tetraphenylphosphonium promotes the ADR resistance in BC^11^. However, the clinical trials of ABCB1 inhibitors are discouraged^5,12^, and the exploration of upstream mechanisms may provide novel insight to ABCB1-mediated ADR resistance in BC.

Long noncoding RNAs (LncRNAs) are transcripts longer than 200 nucleotides and cannot code proteins. Aberrant lncRNA expression is implicated in various biological functions in tumor progression as well as the tumor chemoresistance^13^. LncRNA *A1BG-AS1* is reported to facilitate BC tumor growth and malignant cell behaviors by binding with miR-485-5p to regulate (flotillin-1) FLOT1 expression^14^. A study also suggests that *A1BG-AS1* is a prognostic factor for the 5-year recurrence-free survival of BC patients^15^. However, its role in BC chemoresistance remains unclear.

Insulin-like growth factor 2 mRNA-binding protein 2 (IGF2BP2) belongs to an evolutionally conserved family of RNA-binding proteins, including IGF2BP1, IGF2BP2 and IGF2BP3 in human eukaryotic cells. M^6^A readers were reported to be involved in controlling the fate of mRNA, and the IGF2BP2 was associated with methylated mRNA stability^16^. IGF2BP2 had already suggested to be associated with tumor progression through preserving the stemness phenotype in BC^17^. However, little is known about the post-transcriptionally regulation of IGF2BP2 protein.

In this study, we intended to investigate the function and mechanism of *A1BG-AS1* to regulate the ABCB1-mediated BC ADR resistance. We hypothesized that *A1BG-AS1* enhanced ADR resistance in BC by recruiting IGF2BP2 to stabilize ABCB1 in an m6A-dependent manner. The findings of our study may provide clues against chemoresistance in BC.

## Materials and methods

### Cell culture and treatment

Human BC cell lines (MCF-7, MDA-MB-231) and ADR resistant cells (MCF-7/ADR, MDA-MB-231/ADR) were provided by the Ek-Bioscience (Shanghai, China) and incubated in DMEM with 10% FBS, penicillin (100 U/ml) streptomycin (100 μg/ml) at 37 °C with 5% CO_2_. To maintain the drug resistance, MCF-7/ADR and MDA-MB-231/ADR cells were cultured with 1 μg/mL ADR in cell medium^18^. MCF-7/VCR and MDA-MB-231/VCR cells were cultured with 1.5 μg/mL VCR in cell medium.

### Cell transfection

The lentiviral particles of sh-A1BG-AS1-1/2, sh-LINC00052-1/2, sh-LINC00494-1/2, sh-DSCR8-1/2 and sh-IGF2BP2-1/2 were designed and purchased from GenePharma (Shanghai, China). To generate the lentiviruses, shRNA plasmids were co-transfected into MDA-MB-231 and MCF-7 cells along with envelope (VSVG) and packaging (pGag/Pol, pRev) plasmids using lipofectamine 2000 (Invitrogen). The viral supernatants were harvested and filtered after 48 h transfection. Cells were infected in the presence of a serum-containing medium supplemented with 8 μg/mL polybrene. Following infection for 48 h, cells were selected with 2.0 μg/mL puromycin (Sigma, USA). Knockdown efficiencies were examined by qRT-PCR. Plasmid vector pcDNA3.1-A1BG-AS1, pcDNA3.1-ABCB1 and pcDNA3.1-IGF2BP2 were designed and synthesized by Genepharma via standard molecular cloning approaches. The empty pcDNA3.1 vector was used as a control. Cells were transfected with indicated plasmids using Lipofectamine 3000 and harvested after 48 to 72 h.

### qRT-PCR

RNA extraction was performed with TRIzol reagent (Thermo Fisher, USA) and the cDNAs were synthesized via reverse transcription with HiScript 1st Strand cDNA Synthesis Kit (Vazyme, Nanjing, China). The PCR analysis was conducted using a SYBR Green PCR kit (TaKaRa, Dalian, China) was used to conduct on Applied Biosystems 7500. Gene expression was calculated with the 2^−ΔΔct^ method normalized to GAPDH. The primer sequences were shown as following: *A1BG-AS1*: F: 5′-TTTAGTAGAGACGGGGTTTCGTC-3′, R: 5′-CTGATGGTTGCAAAGGAGTTTG-3; *IGF2BP2*: F: 5′-GTCCTACTCAAGTCCGGCTAC-3′, R: 5′- CATATTCAGCCAACAGCCCAT-3; *ABCB1*: F: 5′-AGGCCAACATACATGCCTTC-3′, R: 5′-CCACCAGAGAGCTGAGTTCC-3; *GAPDH*: F: 5′-TCATTTCCTGGTATGACAACGA-3′, R: 5′-GTCTTACTCCTTGGAGGCC-3.

### Western blot

The total protein in BC cells were extracted by radioimmunoprecipitation assay (RIPA) lysis buffer (Beyotime, Shanghai, China). A bicinchoninic acid (BCA) protein assay kit (Vazyme) was used to determine the protein concentration. After boiling in sample buffer, the protein samples were loaded on 10% sodium dodecyl sulfate–polyacrylamide gel electrophoresis (SDS–PAGE) and electro-transferred to PVDF membranes (Sigma-Aldrich). 5% non-fat milk was applied to block the membranes that were then cultured with the primary antibodies, anti-ABCB1 (ab129450, 1:1000), anti-IGF2BP2 (ab124930, 1:2000) overnight at 4 °C with GAPDH as the loading control. Subsequently, the membranes were cocultured with the secondary antibodies (Horseradish peroxidase-conjugated anti-mouse) at ambient temperature for 2 h. The protein bands were visualized with ECL reagent (Tanon, Shanghai, China) and the protein density was measured with the ImageJ software.

### Subcellular fraction extraction

The nuclear and cytoplasmic fraction of cells was isolated using the PARIS™ kit (Cat#AM1921, Ambion, USA). About 1 × 10^7^ cells were washed with PBS on ice followed by centrifugation at 500× g for 5 min. Cell pellets were resuspended in 500 μl cell fraction buffer, incubated on ice for 10 min, and then centrifuged at 500× g and 4 °C for 5 min to separate the nuclear and cytoplasmic cell fractions. Nuclear pellets were homogenized with the cell disruption buffer. QRT-PCR was used to analyze the cytoplasm and nucleus RNA extract, where GAPDH and U6 were used as normalization controls, respectively.

### RNA stability analysis

The RNA stability was measured as previously described^19^. The actinomycin D (ActD, 5 μg/ml, Sigma-Aldrich) was added to transfected BC cells to block RNA synthesis. Then the RNA expression was assessed at 0, 6, 12, 18, 24 h using qRT-PCR analysis.

### Cell viability

The ADR resistance and viability of BC cells was measured using CCK-8 assays. Parental cells (MCF-7 and MDA-MB-231) and cells treated with ADR (MCF-7/ADR and MDA-MB-231/ADR) were grown into 96-well plates (5000 cells per well). After the treatment of different concentrations of ADR (0, 0.1, 1, 10, 100 μg/mL) and incubation for 48 h. Then 10 μL CCK-8 solution was added into each well and incubated for another 4 h. For transfected cells, at 0, 24, 48, 72, and 96 h post-transfection, MCF-7 and MDA-MB-231 cells viability was assessed by CCK-8 assay. The absorbance at 450 nm was determined using a microplate reader.

### Colony formation

Colony formation assay was conducted for the proliferation evaluation of BC cells. The cells were grown in six-well dishes at 1 × 10^3^ cells/well. After culturing for 14 days, methanol was used to fix the colonies for ten minutes followed with 0.1% crystal violet staining for twenty minutes. Cell colonies were counted manually and imaged under a microscope.

### Transwell

Cell migration ability was subject to a Transwell assay. The transfected cells were suspended in serum-free RPMI 1640 medium and added into the upper transwell chambers. The lower chambers were supplemented with RPMI 1640 medium with 10% FBS. After incubating for 24 h, the methanol was used to fix the migrated cells for ten minutes followed with 0.5% crystal violet staining. A light microscope was used to observe and photograph the migrated cells.

### Flow cytometry

BC apoptosis was analyzed as described in a previous study^20^. Briefly, ADR was added, with the final concentration of 1 μg/ml for MCF-7 for 48 h. 72 h after the transfection as described above, the cells were subject to trypsin with no EDTA. Then the cells were centrifuged at 1500 rpm for 5 min. The supernatant was removed, and 5 ul Annexin V-fluorescein isothiocyanate (FITC) and 5 ul propidine iodide solution (PI, Solarbio, China) was supplemented to further culture for 20 min at ambient temperature in a dark room. Finally, the apoptosis was measured using a flow cytometer (BD Biosciences, USA).

### RNA immunoprecipitation (RIP) assay

An EZ-Magna RIP RNA-Binding Protein Immunoprecipitation Kit (Millipore, USA) was used to perform RIP experiments. After lysing the BC cells with RIP lysis buffer, the cell lysis was cultured with magnetic-bead-coated with 4 μl targeted protein antibodies or control anti-Immunoglobulin G (IgG) at 4 °C overnight. Next, the RNA–protein complex was washed by washing buffer and cultured with proteinase K (Sangon, China) with shaking for 1.5 h to remove proteins, the immunoprecipitated RNAs were then eluted, purified, and measured with qRT-PCR analysis. Antibodies included anti-FBL (ab226178, Abcam, USA), anti-RBM10 (18,012, CST, USA), anti-IGF2BP2 (14672S, CST, USA), anti-ELAVL1 (12,582, CST, USA), anti-NOP58 (ab236724, Abcam, USA), and anti-IgG (ab48386, Abcam, USA).

### Methylated RNA immunoprecipitation (Me-RIP) assay

Trizol reagent (Thermo Fisher) was used to isolate total RNA from BC cells and mRNA was further purified via polyATtract mRNA Isolation Systems (Promega Corp). Then the extracted RNAs were added to MeRIP buffer (150 mM NaCl, 10 mM Tris–HCl, pH 7.5, 0.1% NP-40) and incubated with magnetic beads coated with IgG and anti-m6A (ab151230, Abcam, USA) antibodies for 1 h in order for binding. Next, the purified mRNAs and the complex was added into the buffer with ribonuclease inhibitor and protease inhibitor and maintained overnight at 4 °C. Finally, RNAs bound to m6A was eluted with elution buffer, and purified with phenol–chloroform. RNA expression was subject to qRT-PCR.

### RNA pulldown

RNA pulldown experiments were used to explore the binding of IGF2BP2 with *A1BG-AS1* or ABCB1 in BC cells using a Magnetic RNA Protein Pull-Down Kit (Pierce, USA). Briefly, the biotin-labeled antisense RNA and sense RNA were used to synthesize biotin-labeled *A1BG-AS1* and ABCB1, which were then cultured with the streptavidin beads and cell lysis for 2 h, followed with centrifugation at 4 °C for 5 min. The RNA–protein complex was washed, and measured with western blot.

### Fluorescence in Site Hybridization (FISH)

A FISH detection kit (RiboBio, Guangzhou, China) including probes for *A1BG-AS1* and IGF2BP2 was used following manufacturer’s protocol. Briefly, the treated BC cells were grown on glass bottom cell culture dish followed with 4% paraformaldehyde treatment for ten minutes. After 0.5% Trito X-100 treatment, the cells were blocked with prehybridization buffer and blocking buffer for 30 min at 37 °C. Then the cells were washed with PBS and the pre-hybridization mixture was cleaned. The prepared probes were added and incubated overnight. The nuclei were stained with DAPI for 15 min. Finally, images were taken under a confocal microscope.

### Xenograft mouse models

The procedures of animal study were approved by the Ethics Committee of Tianjin Fifth Central Hospital (No. 2019BWK2007). Sixteen BALB/C nude mice (4–6 weeks, 16–18 g) were provided by the Vital River Laboratory Animal Technology Co., Ltd. (Beijing, China) and divided into the sh-NC and sh-A1BG-AS1-1 (n = 8 per group). The xenograft mouse models were established by injecting MCF-7/ADR cells (1 × 10^7^ in 100 μL RPMI 1640 medium) into the mouse right flank. Tumor size was monitored every week. When the average tumor size reached approximately 100 mm^3^, 5.0 mg/kg adriamycin were subsequently subjected through tail vein every other day. Mice were sacrificed after 4 weeks, and tumors were excised. The study complies with the relevant guidelines and regulations. The study is consistent with the Animal Research: Reporting in Vivo Experiments (ARRIVE) Guidelines.

### Immunohistochemistry

The immunohistochemistry experiments were conducted to explore tumor proliferation potential. Briefly, the tumor tissues were collected, immersed in 4% paraformaldehyde, paraffin-embedded and sliced into 5 μm-thick sections. Following xylene dewaxing, gradient ethanol hydration, and antigen high-pressure repair, the tissue sections were stained with primary antibody against Ki67 (ab16667, 1/200, Abcam) and cultured at 37 °C for 1 h. Next, the secondary antibodies was added and cultured for 30 min. After DAB staining, a microscope was used to observe the images. The positive results were brown in color, and calculated with the Image-Pro Plus 6.0 software.

### Statistical analysis

The statistical analyses were performed with the GraphPad Prism 8.0 Software. The results were expressed as the mean ± S.D. Student’s t-test was used to analyze the statistical difference between two groups, and one way ANOVA was performed for multiple group comparison. A *p* value < 0.05 was considered as statistically significant.

### Ethics approval and consent to participate

The procedures of animal study were approved by the Ethics Committee of Tianjin Fifth Central Hospital (No. 2019BWK2007). The contents of this study are under full compliance with government policy and the Declaration of Helsinki.

## Results

### The expression of *A1BG-AS1* and ABCB1 was positively correlated in BC

**Figure 1:**
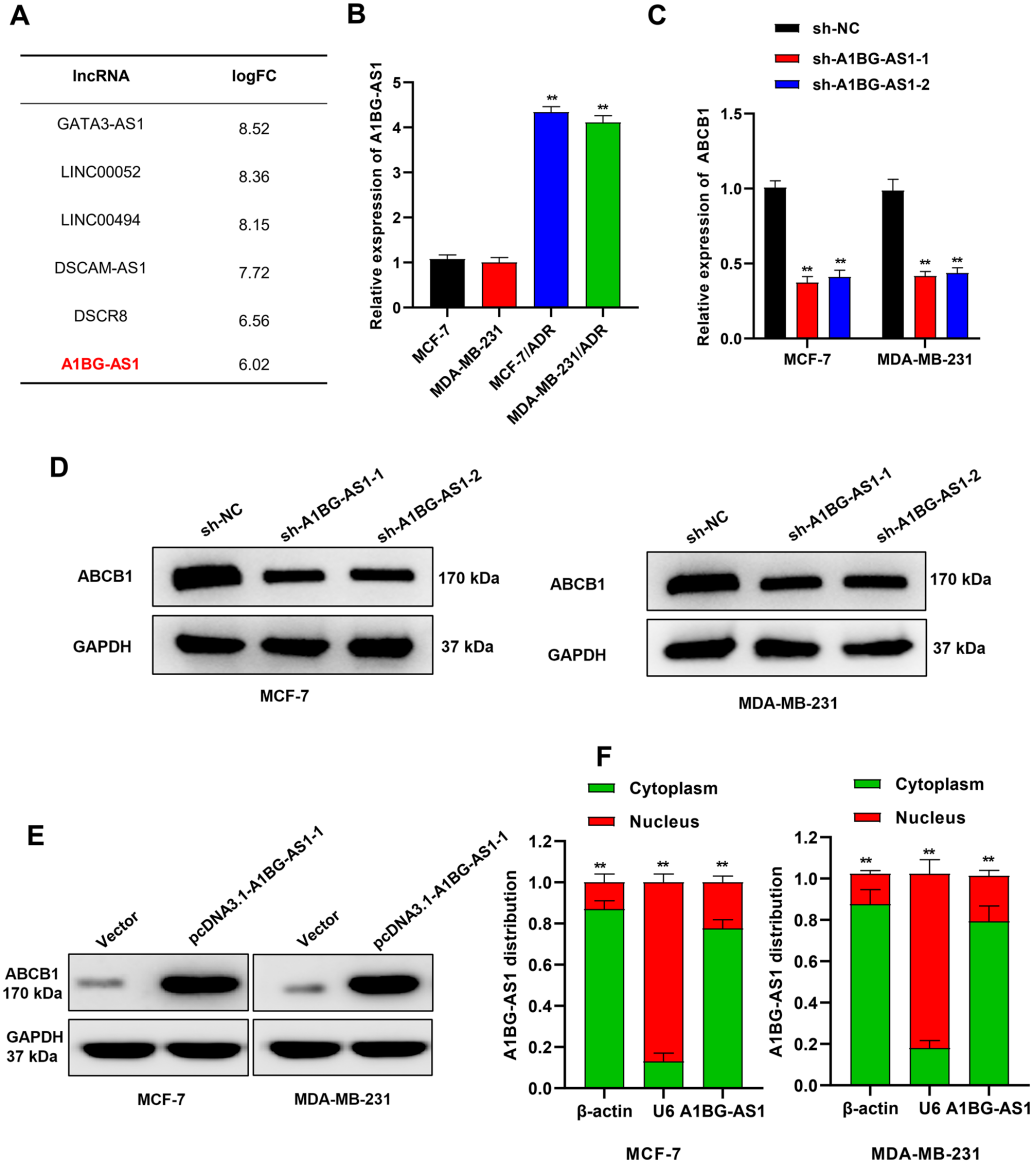
The expression correlation of *A1BG-AS1* and ABCB1 in BC. (**A**) The GSE155478 database (*p* < 0.5, logFC > 6) was used to select lncRNAs highly expressed in MCF-7/ADR cells compared with MCF-7 cells. (**B**) qRT-CPR was used to analyze *A1BG-AS1* expression in BC cell lines (MCF-7, MDA-MB-231) and ADR resistant BC cells (MCF-7/ADR, MDA-MB-231/ADR). (**C**) ABCB1 mRNA levels in *A1BG-AS1* silenced BC cells. (**D**) ABCB1 protein expression in BC cells after *A1BG-AS1* silencing. (**E**) Overexpression of *A1BG-AS1* increased ABCB1 protein level in MCF-7 and MDA-MB-231 cells. (**F**) The cellular distribution of *A1BG-AS1* in BC cells was assessed by subcellular fractionation assays. **p < 0.01.

Based on the GSE155478 database (*p* < 0.5, logFC > 6), six lncRNAs (*GATA3-AS1*, *LINC00052*, *LINC00494*, *DSCAM-AS1*, *DSCR8*, *A1BG-AS1*) were screened out to be highly expressed in MCF-7/ADR cells compared with MCF-7 cells (Fig. 1A). Among the six lncRNAs, the implication of *GATA3-AS1* and *DSCAM-AS1* in BC chemotherapy resistance has been demonstrated in the previous studies^21,22^. Then we analyzed whether lncRNA silencing affected *ABCB1* expression. The silencing efficiency of four lncRNAs was verified using qRT-PCR (Figure S1A). The result showed that *ABCB1* was most significantly downregulated after *A1BG-AS1* knockdown in BC cells (Figure S1B). Moreover, we found the upregulation of *A1BG-AS1* in breast invasive carcinoma on the UALCAN website (Figure S1C). The expression of *A1BG-AS1* in BC cell lines (MCF-7, MDA-MB-231) and BC cells resistant to ADR (MCF-7/ADR, MDA-MB-231/ADR) was detected. *A1BG-AS1* was revealed to be significantly upregulated in ADR resistant BC cells (Fig. 1B). Moreover, *ABCB1* levels were downregulated in BC cells with silenced *A1BG-AS1* (sh-*A1BG-AS1*-1/2) (Fig. 1C). The ABCB1 protein expression was also reduced in BC cells with silenced *A1BG-AS1* (Fig. 1D). In addition, increased *A1BG-AS1* upregulated ABCB1 protein level (Fig. 1E), which indicated that *A1BG-AS1* positively regulated ABCB1 expression in BC cells. Then we detected the cellular distribution of *A1BG-AS1* in BC cells and found its primary distribution in the cytoplasm (Fig. 1F).

### *A1BG-AS1* silencing alleviated the BC ADR resistance

**Figure 2:**
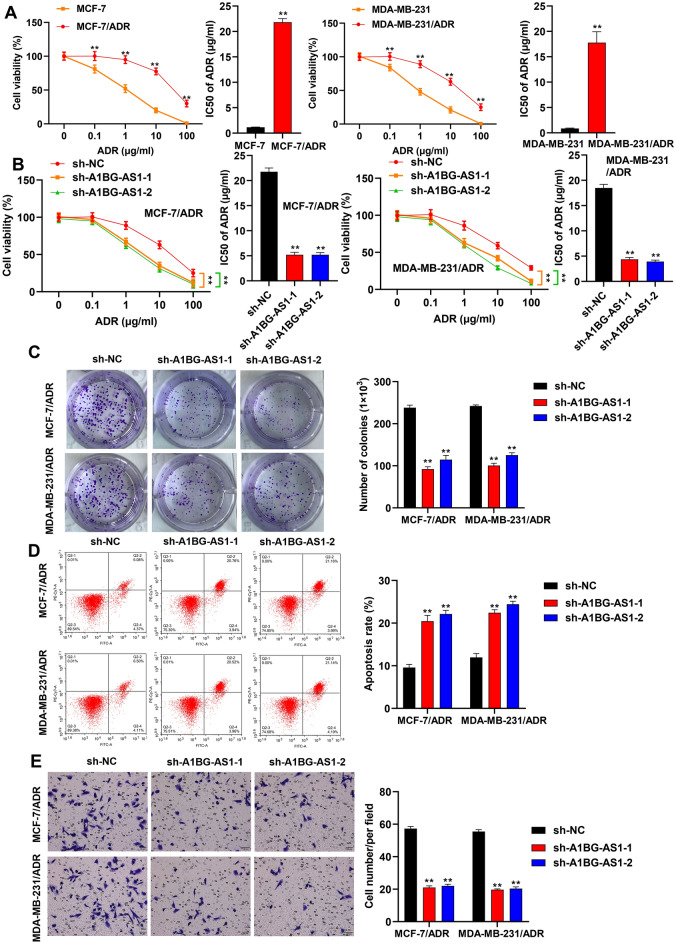
*A1BG-AS1* promoted the ADR resistance of BC. (**A**) The viability and IC50 ADR value of MCF-7 and MDA-MB-231 cells with ADR treatment in different concentration (0, 0.1, 1, 10, 100 μg/mL). (**B**) The viability and IC50 ADR value of ADR resitant BC cells transfected with sh-*A1BG-AS1*-1 and sh-*A1BG-AS1*-2. (**C**) The effect of *A1BG-AS1* silencing on the proliferation potential of ADR resistant BC cells was analyzed using colony formation assays. (**D**) Flow cytometry analysis was performed to evaluate cell apoptosis rate after *A1BG-AS1* deficiency. (**E**) Transwell assays were applied for cell migration assessment. **p < 0.01.

The role of *A1BG-AS1* in ADR resistance of BC was further explored. As shown in Fig. 2A, the viability of MCF-7 and MDA-MB-231 cells was significantly inhibited after ADR treatment in gradient concentration, and the viability of MCF-7/ADR and MDA-MB-231/ADR cells was higher than the cells without ADR resistance. The IC50 ADR value of ADR resistant BC cells were over ten folds than that of BC cells. After *A1BG-AS1* silencing, we first assessed the function of *A1BG-AS1* in parent cells and revealed that knocking down *A1BG-AS1* significantly reduces cell activity of MCF-7 and MDA-MB-231 (Figure S3B). Consistent with this, the viability and IC50 ADR value of cells resistant to ADR was significantly reduced in comparison with the control groups (Fig. 2B). The data of colony formation assay suggested that cell proliferation potential of ADR resistant BC cells was inhibited after *A1BG-AS1* silencing (Fig. 2C). The flow cytometry showed increased apoptosis rate in MDA-MB-231/ADR and MCF-7/ADR cells with *A1BG-AS1* deficiency (Fig. 2D). Moreover, the migration assay indicated that *A1BG-AS1* deficiency showed suppressive effect on the migration ability of ADR resistant BC cells (Fig. 2E).

### IGF2BP2 is a shared RNA binding protein for *A1BG-AS1* and ABCB1 mRNA

**Figure 3:**
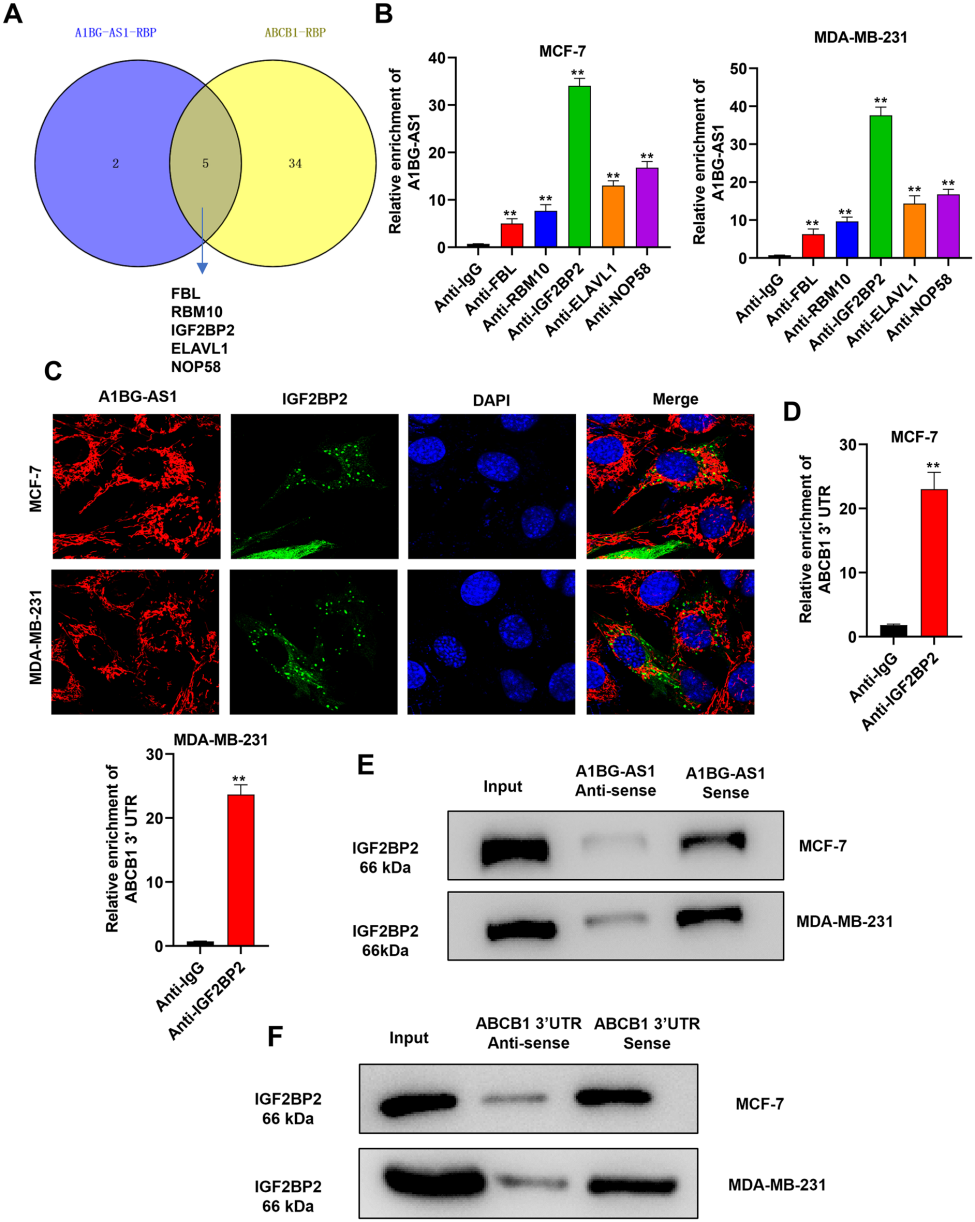
IGF2BP2 is a shared RBP for *A1BG-AS1* and ABCB1 mRNA. (**A**) A venn diagram of shared RBPs (FBL, RBM10, IGF2BP2, ELAVL1, NOP58) for *A1BG-AS1* and ABCB1. (**B**) The RIP assays were applied for the interaction exploration of *A1BG-AS1* and five selected RBPs. (**C**) FISH assays were applied to observe the colocalization of *A1BG-AS1* and IGF2BP2. (**D**) The binding relation of ABCB1 and IGF2BP2 was analyzed by RIP assays. (**E**,**F**) RNA pulldown assay was used for interaction exploration of IGF2BP2 and *A1BG-AS1* or ABCB1. **p < 0.01.

The regulatory mechanism of *A1BG-AS1* on *ABCB1* was investigated. LncRNAs are reported to recruit RNA binding protein (RBP) to stabilize the expression of downstream mRNAs, which mainly occurs in the cytoplasm^23^. The RBPs for *A1BG-AS1* was predicted on the starBase with Cluster ≥ 10, and RBPs for *ABCB1* mRNA was predicted under the condition of CLIP-DaTa ≥ 2 (Figure S2A). A total of 5 shared RBPs (FBL, RBM10, IGF2BP2, ELAVL1, NOP58) were found (Fig. 3A). Then RIP assays were used for the interaction evaluation of *A1BG-AS1* and five selected RBPs. *A1BG-AS1* was most abundantly enriched in the precipitates of anti-IGF2BP2 in MDA-MB-231 and MCF-7 cells (Fig. 3B). According to the results of FISH assays, we found the colocalization of *A1BG-AS1* and *IGF2BP2* in the cytoplasm of parental BC cells (Fig. 3C). FISH assay with probe also showed that *A1BG-AS1* and *IGF2BP2* were mainly distributed in cytoplasm of ADR-resistant cells (Figure S3A), suggesting that there are no differences between the parental and ADR-resistant BC cells of *A1BG-AS1* and *IGF2BP2* distribution. As previously reported, IGF2BP2 is a N6-methyladenosine (m6A) reader that can regulate the mRNA stability^24^. Moreover, the SRAMP database predicted that *ABCB1* 3’UTR possesses m6A modification site with very high confidence (Figure S2B). We further analyzed the interaction of *ABCB1* and *IGF2BP2* using RIP assays, which showed that *ABCB1* 3’UTR was significantly enriched in the precipitates of anti-IGF2BP2 in BC cells (Fig. 3D). RNA pulldown assays were performed to further analyze the interaction of IGF2BP2 and *A1BG-AS1* or *ABCB1*. The results demonstrated that IGF2BP2 was abundantly enriched in the complex pulldown by *A1BG-AS1* and *ABCB1*, which further proved that *A1BG-AS1* and *ABCB1* could combine with IGF2BP2, respectively (Fig. 3E,F). Overall, IGF2BP2 serves as a shared RBP for *A1BG-AS1* and ABCB1 mRNA.

### *A1BG-AS1* recruited IGF2BP2 to stabilize ABCB1 mRNA expression in an m6Adependent manner

**Figure 4:**
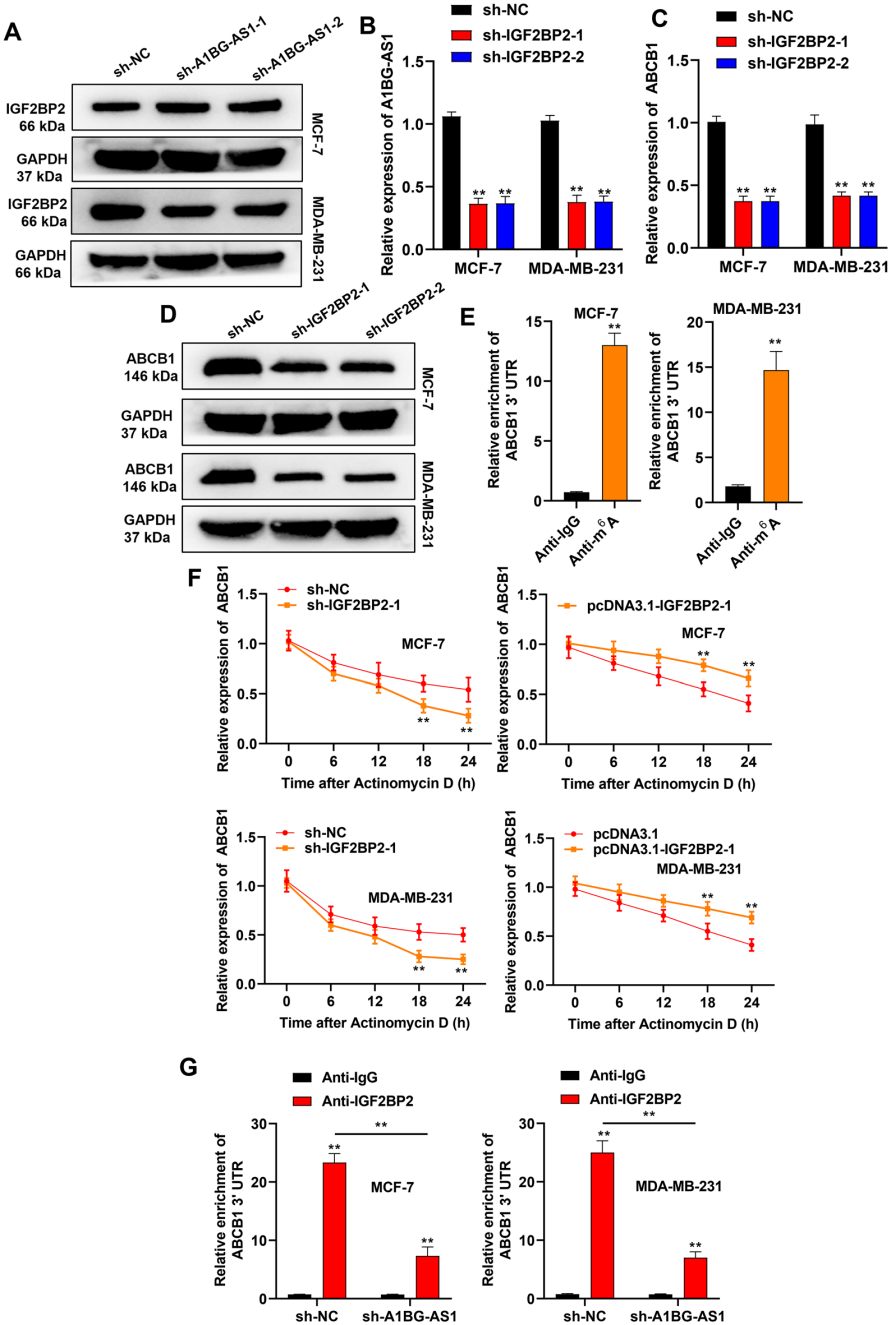
*A1BG-AS1* recruited IGF2BP2 to stabilize ABCB1 mRNA expression in an m6A dependent manner. (**A**) IGF2BP2 protein expression in BC cells with *A1BG-AS1* knockdown. (**B**) *A1BG-AS1* mRNA levels after IGF2BP2 silencing. (**C**,**D**) ABCB1 mRNA and protein levels after IGF2BP2 silencing. (**E**) Me-RIP assays was applied to explore the interaction of ABCB1 3’UTR and anti-m6A. (**F**) BC cells with IGF2BP2 alteration were treated with actinomycin D to block RNA synthesis, and the degradation of ABCB1 mRNA was examined using RT-qPCR assay at different time point. (**G**) The enrichment of ABCB1 3’UTR in the precipitates of anti-IGF2BP2 in breast cancer cells with *A1BG-AS1* silencing. **p < 0.01.

The explicit mechanism of *A1BG-AS1* to regulate IGF2BP2 was further explored. The effect of *A1BG-AS1* silencing on IGF2BP2 protein expression was evaluated using western blot. As shown in Fig. 4A, *A1BG*-*AS1* knockdown did not significantly affect the IGF2BP2 protein levels. The knockdown efficiency of *IGF2BP2* was confirmed using qRT-PCR (Figure S2C). *IGF2BP2* silencing was revealed to significantly reduce the mRNA level of *A1BG-AS1* in BC cells (Fig. 4B). *IGF2BP2* silencing was revealed to significantly reduce the mRNA and protein levels of ABCB1 in BC cells (Fig. 4C,D). Furthermore, the results of Me-RIP assays exhibited that *ABCB1* 3’UTR was significantly enriched in the precipitates of anti-m6A in BC cells, which indicated that *ABCB1* 3’UTR bound with m6A (Fig. 4E). The mRNA stability of *ABCB1* was analyzed in BC cells treated with actinomycin D. As expected, the half-life of *ABCB1* mRNA was dramatically shortened in the situation of deletion of *IGF2BP2*, while the half-life of *ABCB1* mRNA was prolonged by overexpressed *IGF2BP2*. Collectively, these data suggested that *IGF2BP2* enhanced *ABCB1* mRNA stability (Fig. 4F). According to RIP assays, *A1BG-AS1* silencing reduced the enrichment of *ABCB1* 3’UTR in the precipitates of anti-IGF2BP2, which indicated that *A1BG-AS1* promoted the binding of *ABCB1* 3’UTR to *IGF2BP2* (Fig. 4G).

### *A1BG-AS1* promoted the ADR resistance of BC cells by upregulating ABCB1

**Figure 5:**
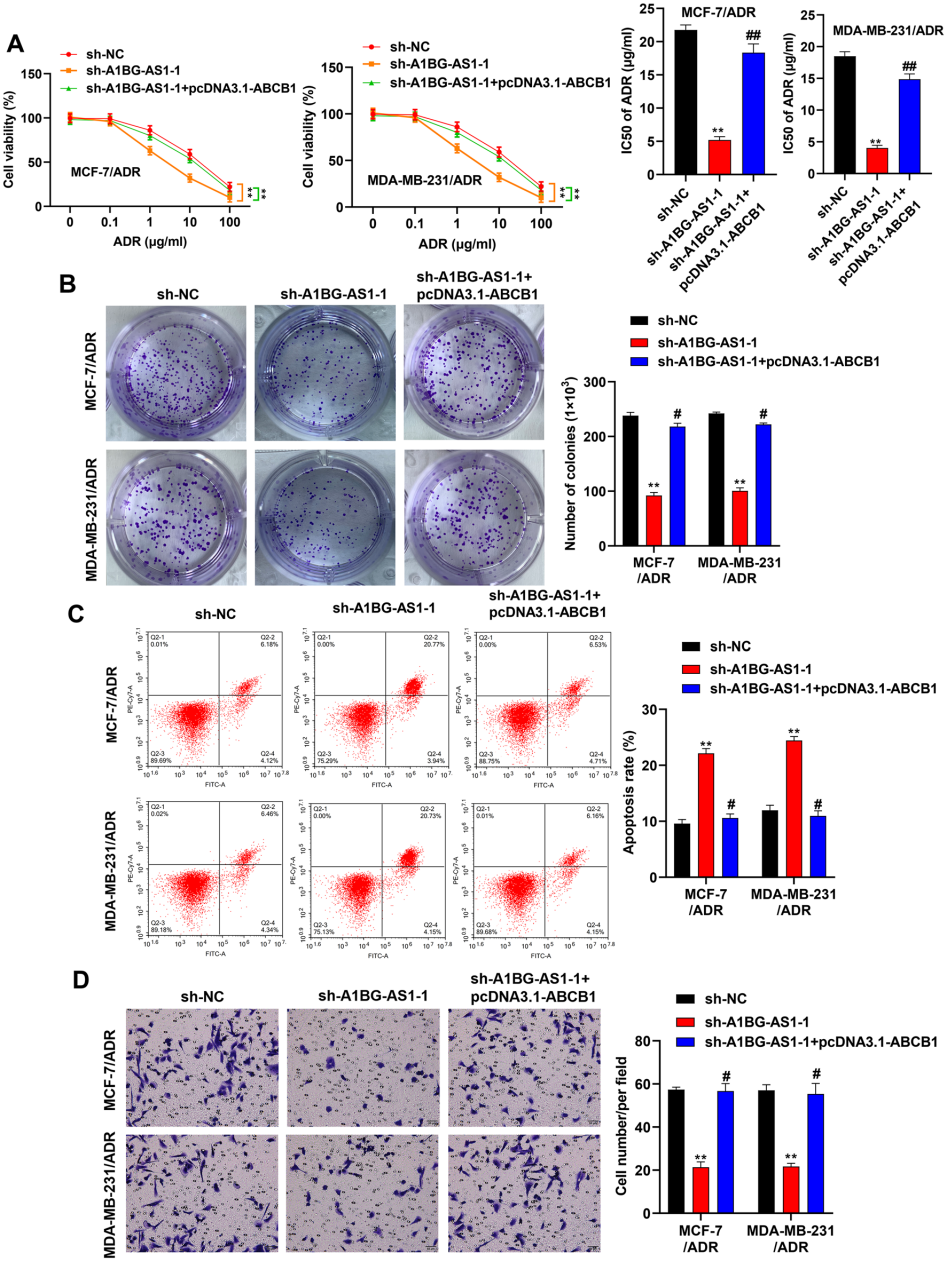
*A1BG-AS1* promoted breast cancer ADR resistance by upregulating ABCB1. (**A**) The viability and IC50 of ADR in ADR resistant BC cells with indicated transfections. (**B**) Cell colony formation potential under indicated treatments. (**C**) BC cell apoptosis was subject to flow cytometry analysis. (**D**) The BC cell migration ability was evaluated by Transwell migration assay. **p < 0.01 versus sh-NC, #*p* < 0.05, ##*p* < 0.01 versus sh-*A1BG-AS1*.

Rescue assays were used to explore the function and mechanism of *A1BG-AS1* for BC ADR resistance. *ABCB1* overexpression efficiency was confirmed in BC cells (Figure S2D). The reduced viability and IC50 of ADR in ADR resistant BC cells with *A1BG-AS1* deficiency was reversed after ABCB1 overexpression (Fig. 5A). It is known that changes in ABCB1 may lead to sensitization to other ABCB1 substrate drugs. Therefore, cell activity was detected in vinblastine-treated MDA-MB-231 and MCF-7 cells transfected with distinct constructures (Figure S3C). The results showed that the reduced viability and IC50 of VCR in VCR resistant BC cells with *A1BG-AS1* deficiency was reversed after ABCB1 overexpression, suggesting that the role of ABCB1 in the above observed is not just unique to ADR. The proliferation potential of breast cancer was inhibited after *A1BG-AS1* knockdown, which was rescued by *ABCB1* upregulation (Fig. 5B). *A1BG-AS1* silencing induced elevation in apoptosis was reversed by the transfection of *ABCB1* in BC cells (Fig. 5C). Moreover, the migration ability of BC cells was suppressed after *A1BG-AS1* silencing and *ABCB1* overexpression reversed this trend (Fig. 5D). Overall, the results indicated that *A1BG-AS1* enhanced the ADR resistance of BC cells by upregulating ABCB1.

### *A1BG-AS1* promoted BC ADR resistance in vivo

**Figure 6:**
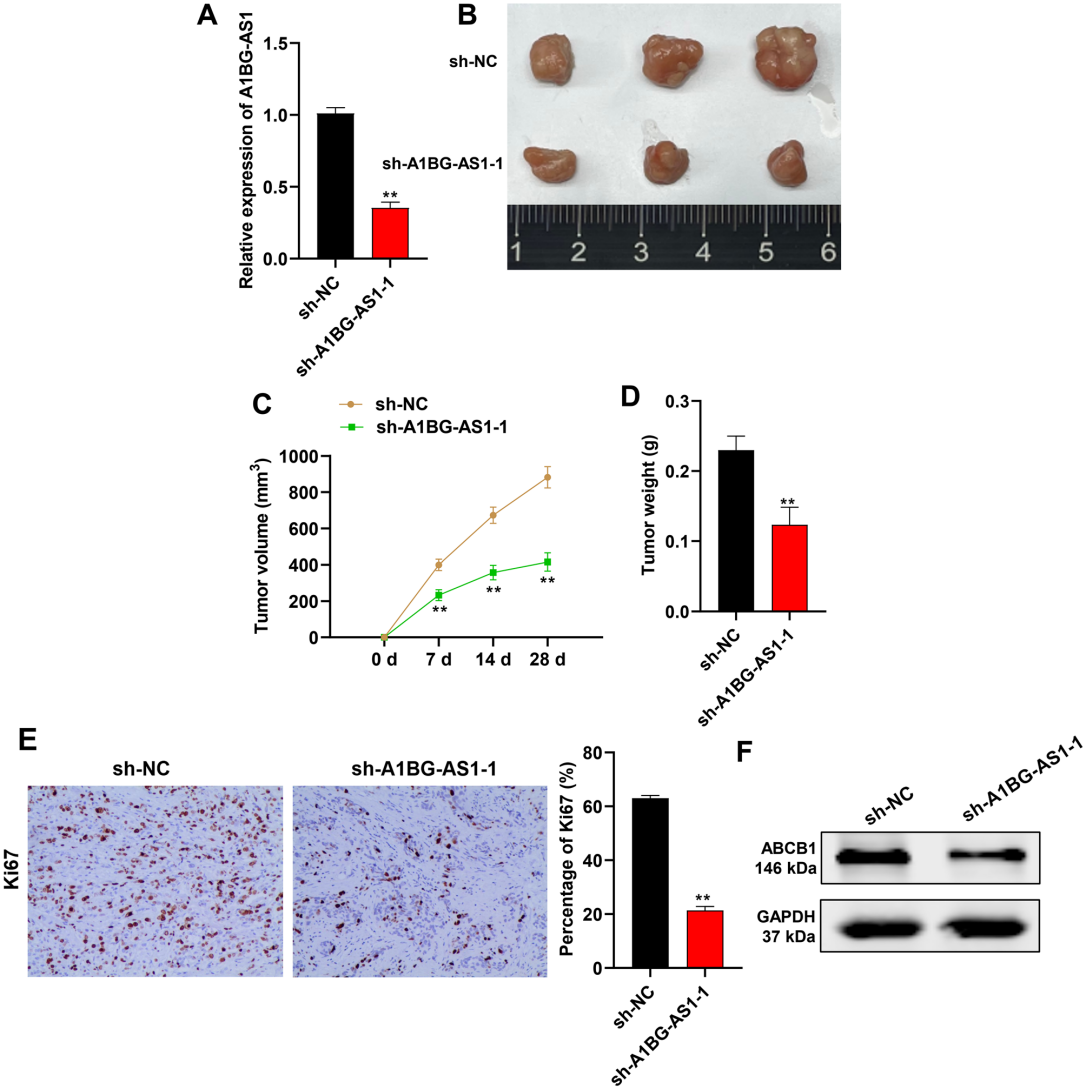
*A1BG-AS1* promoted BC ADR resistance in vivo. (**A**) The qPCR was used to measure the *A1BG-AS1* expression in mouse tumor tissues. (**B**) The images of mouse tumors in the control and *A1BG-AS1* silenced groups. (**C**,**D**) The mouse tumor size and weight in the control and *A1BG-AS1* silenced groups. (**E**) The immunohistochemistry was used to measure the Ki67 expression in mouse tumor tissues. (**F**) Western blot was used to assess the protein expression of ABCB1 in tumor tissues. **p < 0.01.

Xenograft mouse models were established by subcutaneously injecting MCF-7/ADR cells transfected with sh-NC or sh-A1BG-AS1-1. The validation of the tumor xenograft model, qPCR was used to detected *A1BG-AS1* expression in tumor. The results showed that the *A1BG-AS1* expression was significantly decreased after *A1BG-AS1* silencing, which indicated that xenograft model was successfully established (Fig. 6A). The tumor size was smaller in the sh-A1BG-AS1 group in comparison with the control groups (Fig. 6B). The tumor volume was reduced and growth rate was inhibited after *A1BG-AS1* silencing (Fig. 6C). *A1BG-AS1* knockdown was also revealed to reduce the tumor weight than the control groups (Fig. 6D). According to the result of Ki67 immunohistochemistry, the Ki67 expression was significantly decreased after *A1BG-AS1* silencing, which indicated that silenced *A1BG-AS1* suppressed BC tumor growth in vivo (Fig. 6E). ABCB1 protein levels were demonstrated to be reduced after *A1BG-AS1* silencing in mouse tumor tissues (Fig. 6F).

## Discussion

In this study, we found that expression of *A1BG-AS1* was positively correlated with *ABCB1* in BC. *A1BG-AS1* was also found significantly upregulated in the ADR resistant BC cells. As revealed by loss-of-function assays, *A1BG-AS1* knockdown facilitated cell apoptosis and suppressed the proliferation potential as well as the migration abilities of BC cells resistant to ADR. IGF2BP2 served as a shared RBP for *A1BG-AS1* and *ABCB1* mRNA and *A1BG-AS1* recruited IGF2BP2 to stabilize *ABCB1* mRNA expression in an m6Adependent way. Rescue assays further demonstrated that *A1BG-AS1* enhanced the ADR resistance of BC cells by upregulating *ABCB1*. Moreover, we also found that *A1BG-AS1* silencing suppressed the ADR resistance and tumor growth in vivo.

Drug resistance is one of the main obstacles for BC chemotherapy that leads to poor prognosis of patients. Increasing studies have demonstrated that the MDR1/P-gp overexpression leads to cancer cells resistance to lipophilic compounds such as ADR, paclitaxel and vinblastine^25–27^. The regulation of MDR1/P-gp expression is also critical to reverse the chemoresistance of cancer cells. As previously reported, miR-506 inhibits the resistance of colorectal cancer cells to oxaliplatin by downregulating MDR1/P-gp^28^. MiR-27a upregulates MDR1/P-gp levels by targeting HIPK2 in ovarian cancer cells, enhancing the resistance of ovarian cancer cells to paclitaxel^29^. Inhibition of HIF-1α improves the sensitivity of colon cancer cells to multiple drugs by downregulating MDR1/P-gp^30^. MDR1/P-gp is an effective target for multidrug resistance in the therapy of various cancers. In our study, we found *ABCB1* was positively regulated by *A1BG-AS1*, and *ABCB1* overexpression reversed the suppressive effect induced by *A1BG-AS1* knockdown on BC cells with ADR resistance.

LncRNAs are regarded as mediators of chemoresistance that regulates drug efflux, DNA damage as well as the cell apoptosis^31^. The expression of *A1BG-AS1* is predicted to be positively correlated with ABCB1 in BC cells with resistance to ADR, and the regulatory mechanism of *A1BG-AS1* on ABCB1 was investigated. We found IGF2BP2 served as a shared RBP for *A1BG-AS1* and *ABCB1* mRNA. N6-methyladenosine (m6A) modification is reported to regulate cellular biological processes, and IGF2BP2 is known to be an m6A reader to modulate the stability of mRNA transcripts in an m6A-dependent manner^24,32,33^. Considering that *ABCB1* 3’UTR possesses the m6A modification site with high confidence, we analyzed the interaction of *ABCB1* 3’UTR and IGF2BP2 in BC cells. We found that IGF2BP2 bound to *ABCB1* 3’UTR, and IGF2BP2 silencing decreased *ABCB1* mRNA stability and expression. The interaction of IGF2BP2 and *A1BG-AS1* was also investigated. *A1BG-AS1* bound with IGF2BP2 but did not affect IGF2BP2 expression in BC cells. Moreover, we found that *A1BG-AS1* promoted the binding of IGF2BP2 to *A1BG-AS1*. *A1BG-AS1* was demonstrated to stabilize *ABCB1* mRNA expression by recruiting IGF2BP2. There are more directions to explore in this study, for example, we verified that the effect of *ABCB1* is not unique to ADR and can exhibit the same effect in VCR, but the specific molecular mechanism still deserves further exploration. Whether *A1BG-AS1* regulates the sensitivity of BC cells to other chemotherapeutics by recruiting IGF2BP2 to upregulate ABCB1 is also worth studying.

In conclusion, *A1BG-AS1* promotes the ADR resistance of BC tumors and cells by recruiting ABCB1 to stabilize IGF2BP2. The exploration of molecular mechanism in the chemoresistance of BC may provide novel therapeutic targets for anti-cancer treatments.

### Supplementary Information

Supplementary Figures.

Supplementary Figures.

## Data Availability

Datasets analyzed during the current research will be available from corresponding author on reasonable request.
