# Supplementary material for: A1BG-AS1 promotes adriamycin resistance of breast cancer by recruiting IGF2BP2 to upregulate ABCB1 in an m6A-dependent manner
Source: Sci Rep. 2023 Nov 25;13:20730. doi: 10.1038/s41598-023-47956-2 (PMC10676358; doi:10.1038/s41598-023-47956-2)
Supplement: Supplementary file 1 — Supplementary Figures. [file 41598_2023_47956_MOESM1_ESM.pptx]

## Slide 1
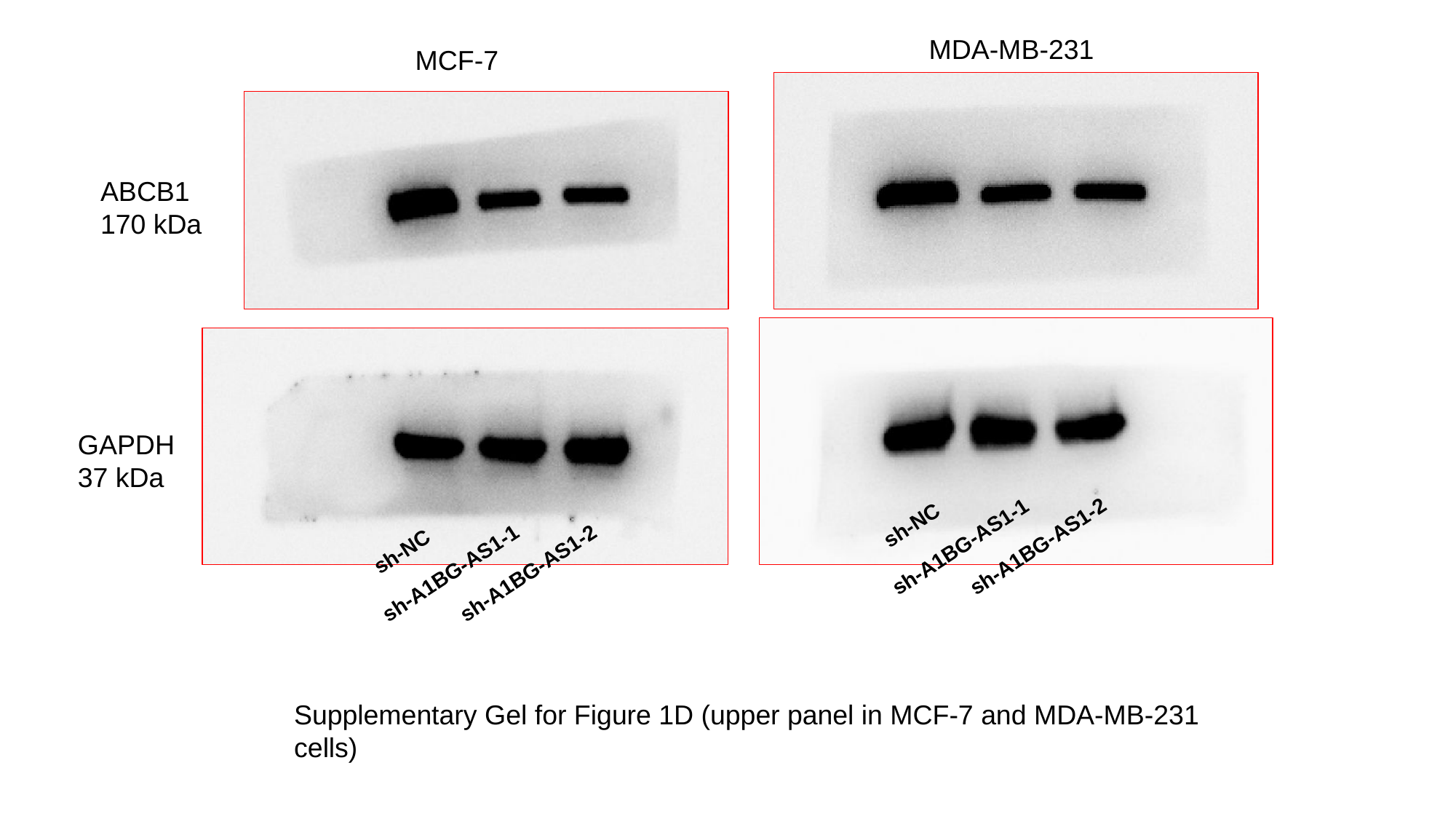

MDA-MB-231
MCF-7
ABCB1
170 kDa
GAPDH
37 kDa
sh-NC
sh-A1BG-AS1-2
sh-A1BG-AS1-1
sh-NC
sh-A1BG-AS1-2
sh-A1BG-AS1-1
Supplementary Gel for Figure 1D (upper panel in MCF-7 and MDA-MB-231 cells)

## Slide 2
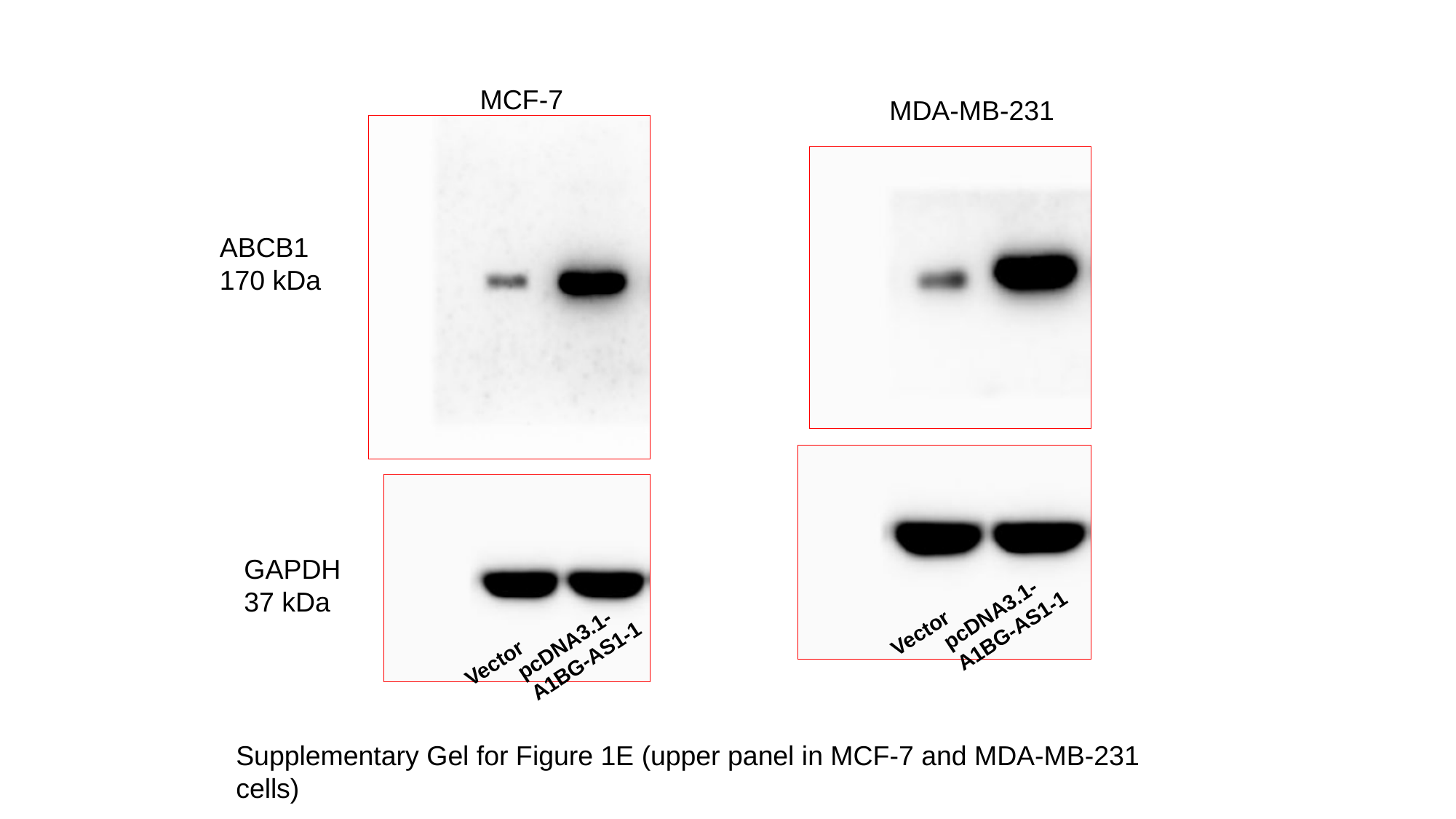

MCF-7
MDA-MB-231
ABCB1
170 kDa
GAPDH
37 kDa
pcDNA3.1-A1BG-AS1-1
Vector
pcDNA3.1-A1BG-AS1-1
Vector
Supplementary Gel for Figure 1E (upper panel in MCF-7 and MDA-MB-231 cells)

## Slide 3
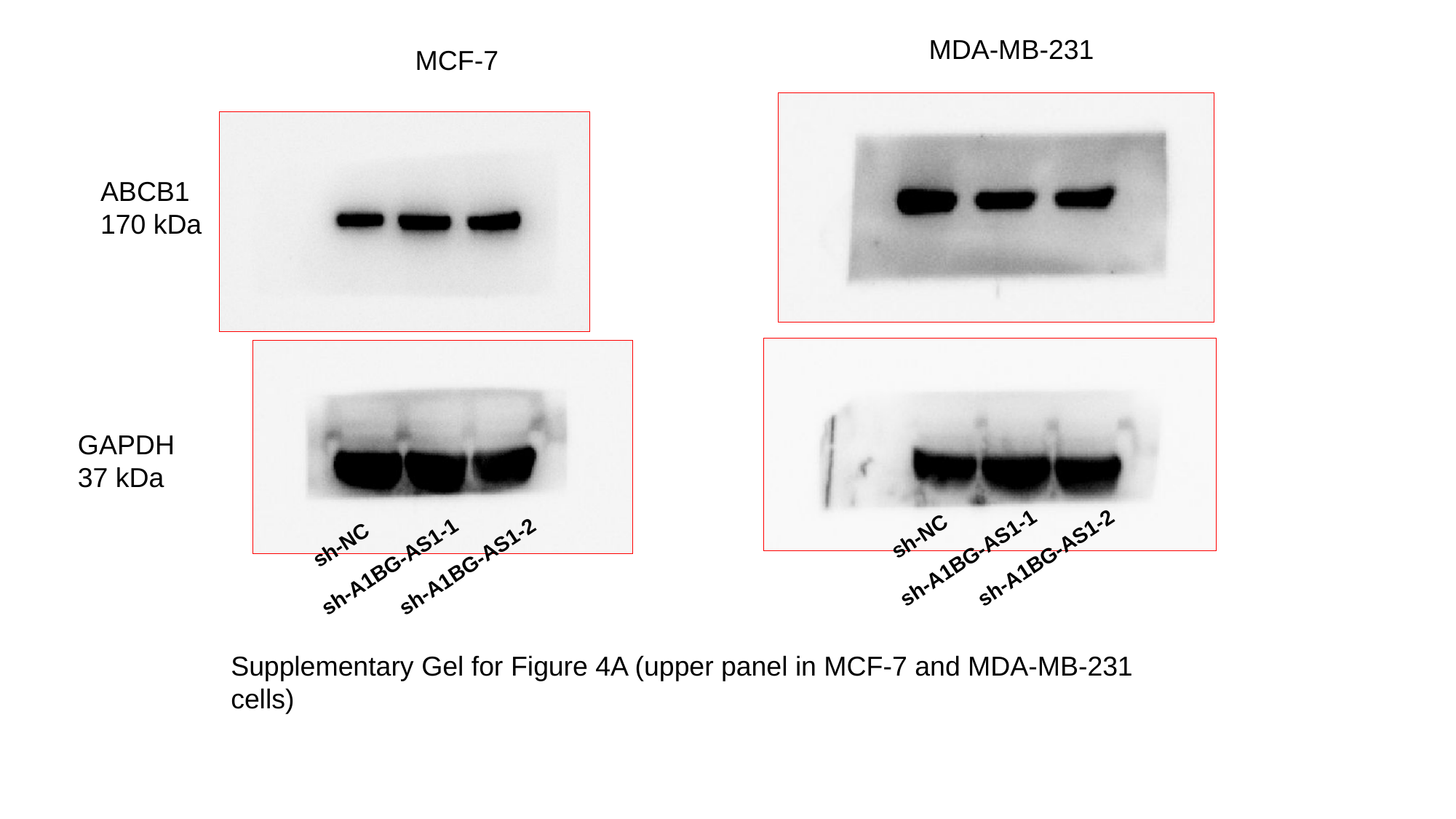

MDA-MB-231
MCF-7
ABCB1
170 kDa
GAPDH
37 kDa
sh-NC
sh-NC
sh-A1BG-AS1-2
sh-A1BG-AS1-1
sh-A1BG-AS1-2
sh-A1BG-AS1-1
Supplementary Gel for Figure 4A (upper panel in MCF-7 and MDA-MB-231 cells)

## Slide 4
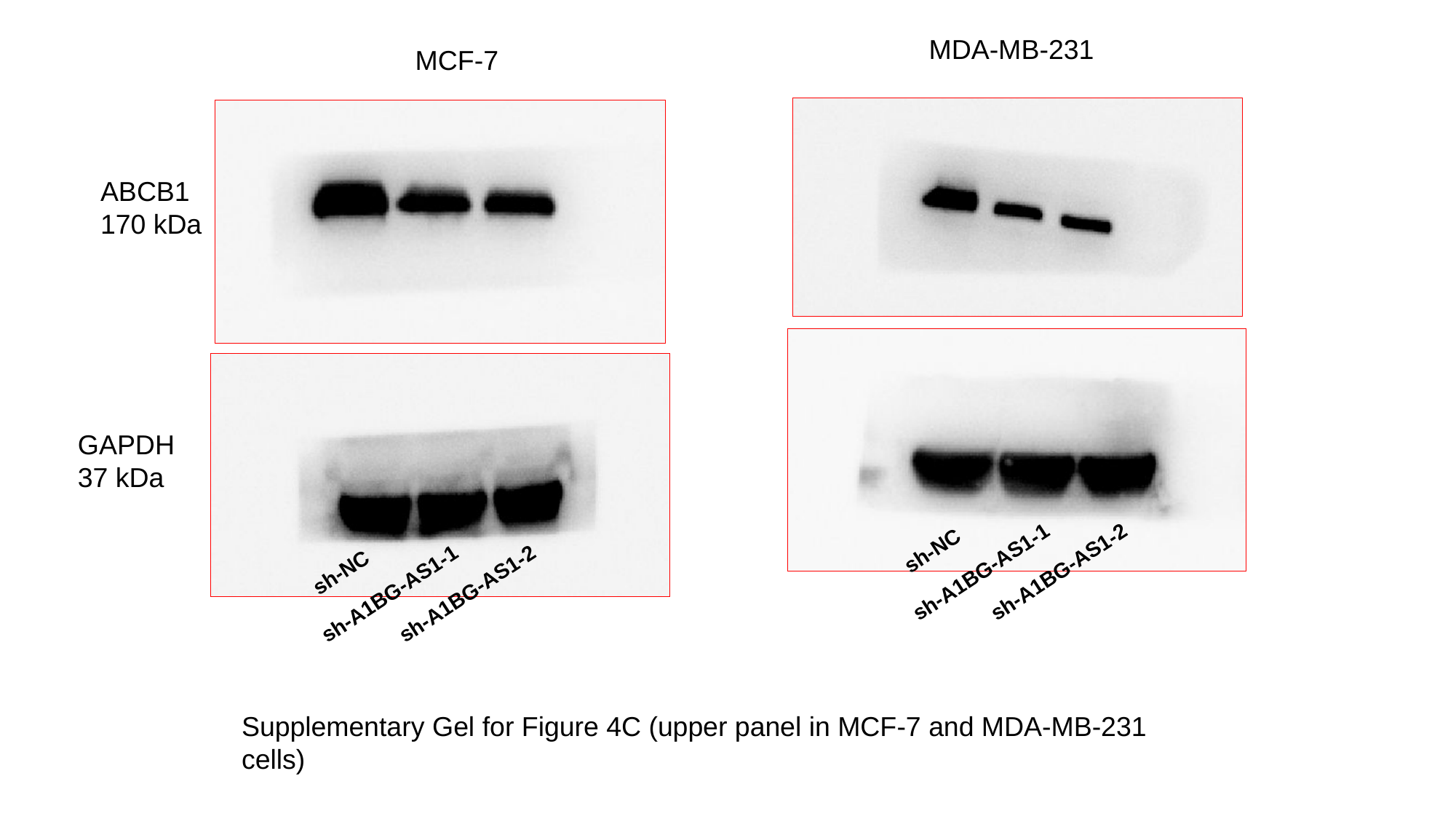

MDA-MB-231
MCF-7
ABCB1
170 kDa
GAPDH
37 kDa
sh-NC
sh-A1BG-AS1-2
sh-A1BG-AS1-1
sh-NC
sh-A1BG-AS1-2
sh-A1BG-AS1-1
Supplementary Gel for Figure 4C (upper panel in MCF-7 and MDA-MB-231 cells)

## Slide 5
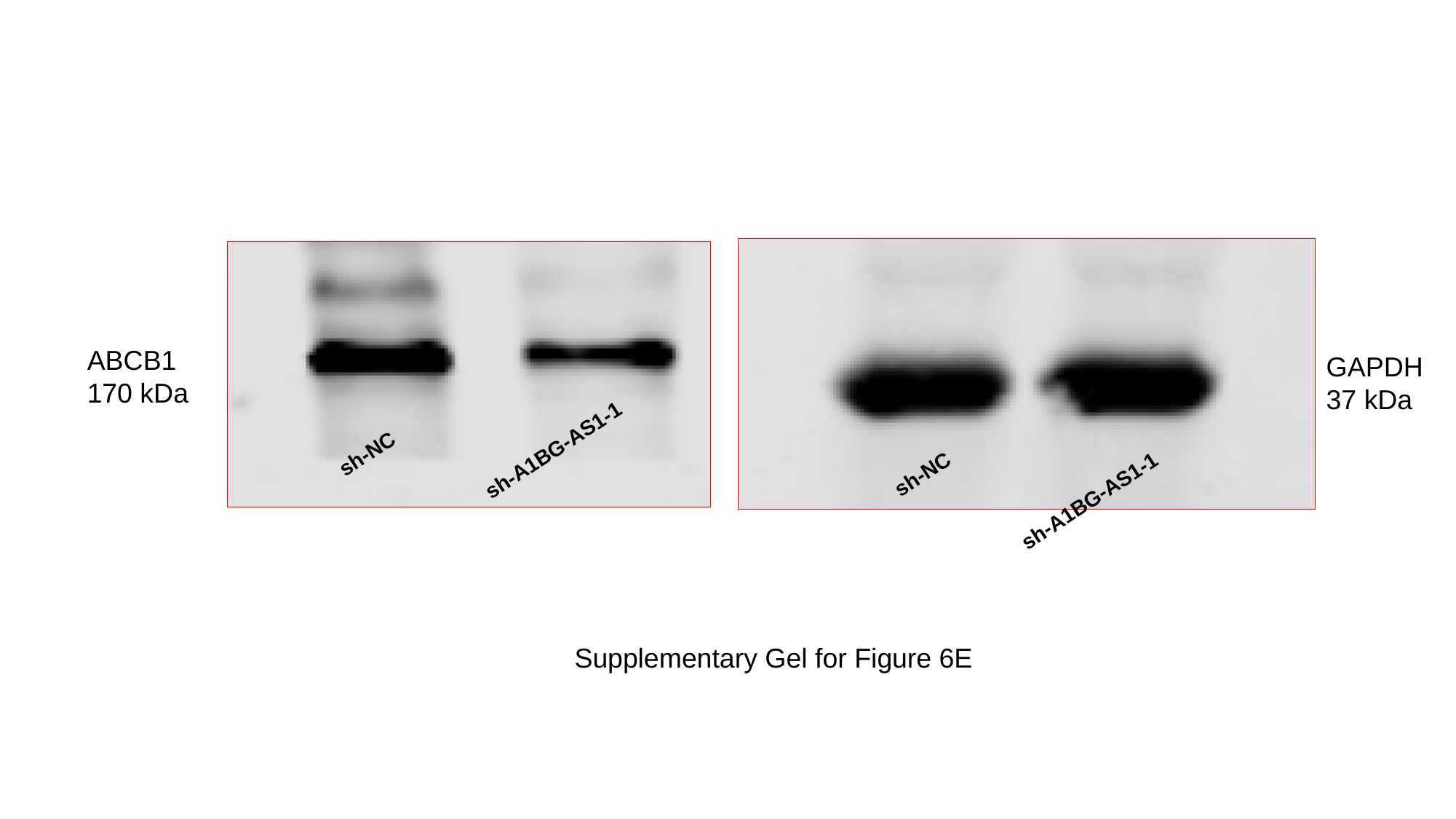

ABCB1
170 kDa
GAPDH
37 kDa
sh-A1BG-AS1-1
sh-NC
sh-NC
sh-A1BG-AS1-1
Supplementary Gel for Figure 6E
