# Supplementary material for: A1BG-AS1 promotes adriamycin resistance of breast cancer by recruiting IGF2BP2 to upregulate ABCB1 in an m6A-dependent manner
Source: Sci Rep. 2023 Nov 25;13:20730. doi: 10.1038/s41598-023-47956-2 (PMC10676358; doi:10.1038/s41598-023-47956-2)
Supplement: Supplementary file 2 — Supplementary Figures. [file 41598_2023_47956_MOESM2_ESM.docx]

**
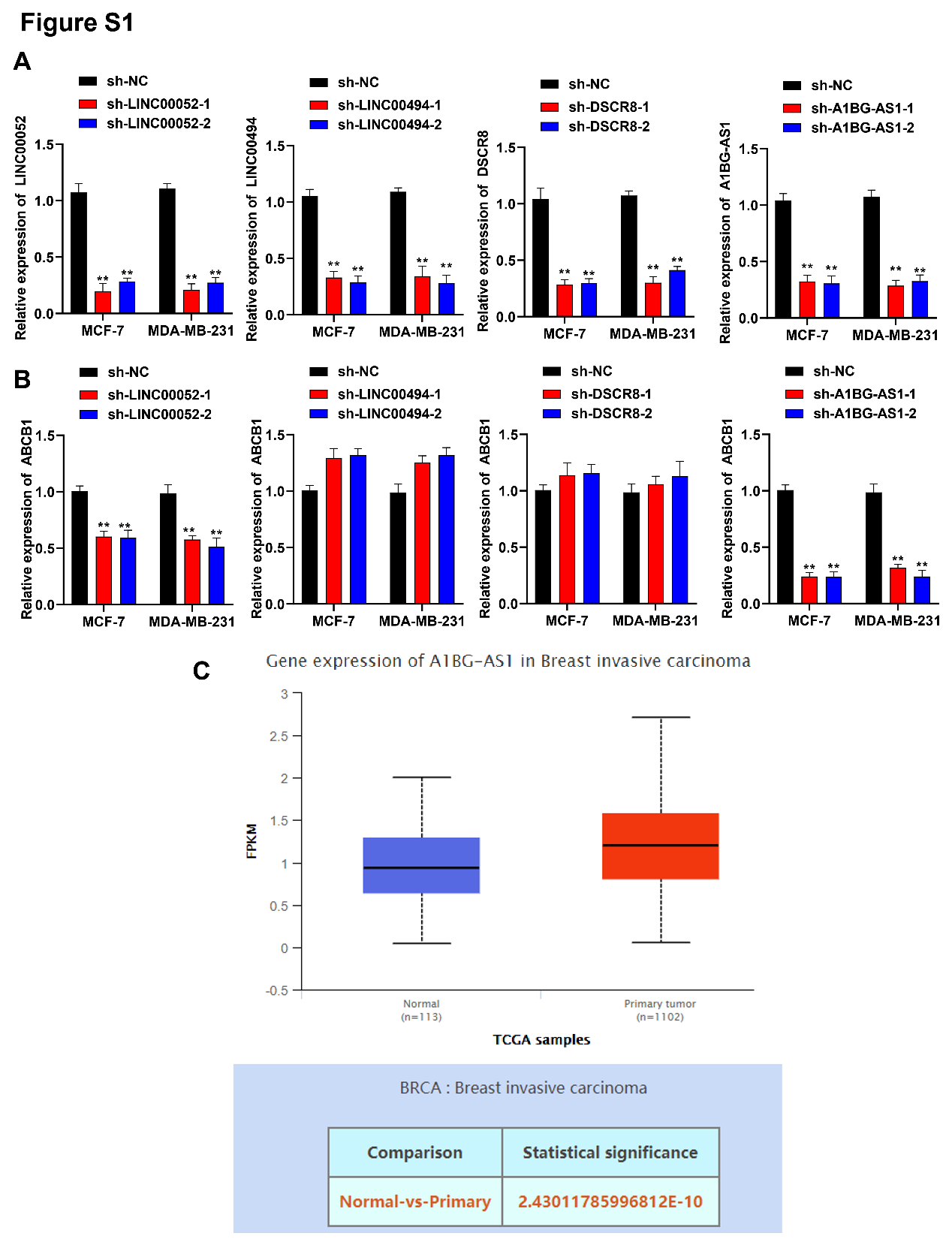
Figure S1. The effect of lncRNAs silencing on *ABCB1* expression.** (A) The knockdown efficiency of *LINC00052*, *LINC00494*, *DSCR8* and *A1BG-AS1*. (B) *ABCB1* levels in breast cancer cells with silenced lncRNAs, respectively. (C) *A1BG-AS1* expression in breast cancer tissues and normal tissues on the UALCAN website ([http://ualcan.path.uab.edu/).**p<0.01](http://ualcan.path.uab.edu/).**p%3c0.01).


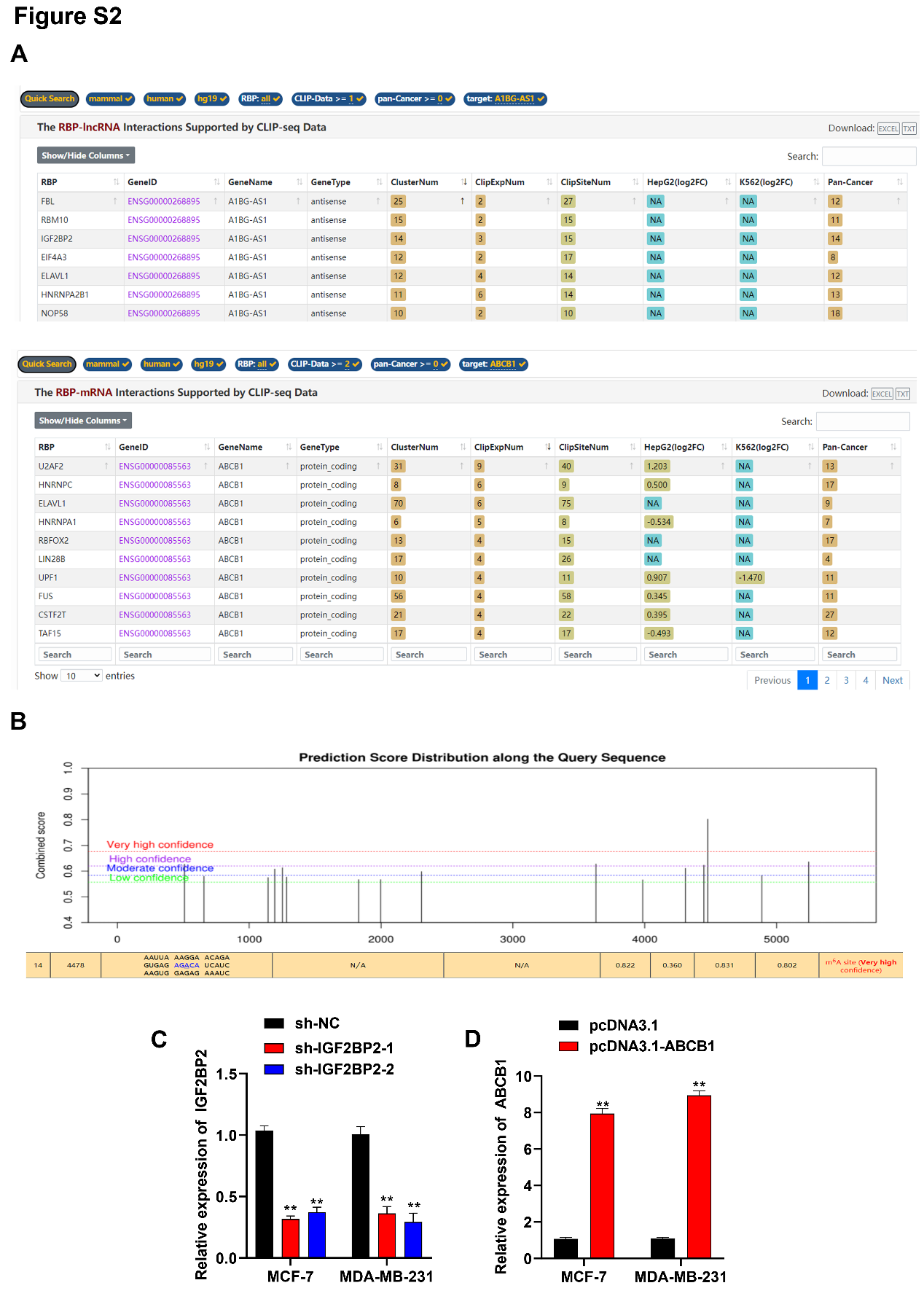


**Figure S2. The shared RBPs for *A1BG-AS1* and *ABCB1*.** (A) The starBase predicted RBPs for *A1BG-AS1* under Cluster≥10, and RBPs for *ABCB1* mRNA under CLIP-DaTa≥2. (B) The SRAMP database (http://www.cuilab.cn/sramp) was used to predict the m6A modification site of *ABCB1* 3’UTR. (C) The knockdown efficiency of *IGF2BP2* in breast cancer cells was detected using qRT-PCR. **p<0.01. (D) *ABCB1* expression in breast cancer cells transfected with pcDNA3.1 or pcDNA3.1/ABCB1 was detected using qRT-PCR. **p<0.01.


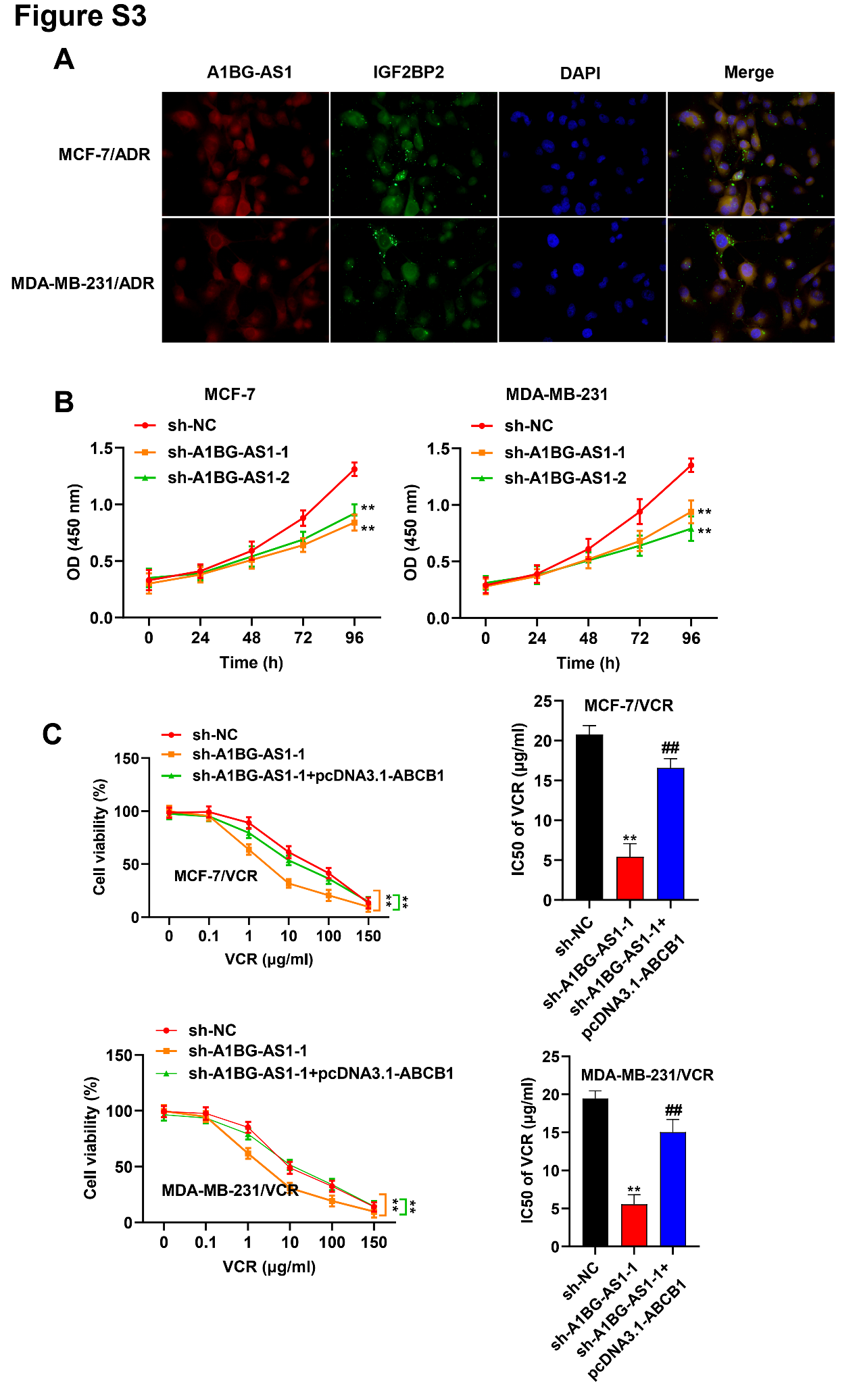


**Figure S3.** (A) FISH assays were applied to observe the colocalization of *A1BG-AS1* in ADR-resistant breast cancer cells. (B) CCK-8 assay was performed to measure the cell viability of MDA-MB-231 cells and MCF-7 cells transfected with sh-NC and sh-A1BG-AS1-1/2. (C) The viability and IC50 of VCR in VCR resistant BC cells with indicated transfections. **p<0.01 vs sh-NC, ##p<0.01 vs sh-*A1BG-AS1*.
